# MOV10 RNA Helicase Is a Potent Inhibitor of Retrotransposition in Cells

**DOI:** 10.1371/journal.pgen.1002941

**Published:** 2012-10-18

**Authors:** John L. Goodier, Ling E. Cheung, Haig H. Kazazian

**Affiliations:** McKusick-Nathans Institute for Genetic Medicine, Johns Hopkins University School of Medicine, Baltimore, Maryland, United States of America; Fred Hutchinson Cancer Research Center, United States of America

## Abstract

MOV10 protein, a putative RNA helicase and component of the RNA–induced silencing complex (RISC), inhibits retrovirus replication. We show that MOV10 also severely restricts human LINE1 (L1), Alu, and SVA retrotransposons. MOV10 associates with the L1 ribonucleoprotein particle, along with other RNA helicases including DDX5, DHX9, DDX17, DDX21, and DDX39A. However, unlike MOV10, these other helicases do not strongly inhibit retrotransposition, an activity dependent upon intact helicase domains. MOV10 association with retrotransposons is further supported by its colocalization with L1 ORF1 protein in stress granules, by cytoplasmic structures associated with RNA silencing, and by the ability of MOV10 to reduce endogenous and ectopic L1 expression. The majority of the human genome is repetitive DNA, most of which is the detritus of millions of years of accumulated retrotransposition. Retrotransposons remain active mutagens, and their insertion can disrupt gene function. Therefore, the host has evolved defense mechanisms to protect against retrotransposition, an arsenal we are only beginning to understand. With homologs in other vertebrates, insects, and plants, MOV10 may represent an ancient and innate form of immunity against both infective viruses and endogenous retroelements.

## Author Summary

LINE1s, the only active autonomous mobile DNA in humans, occupy at least 17% of our genome. It is believed that about 100 L1s are potentially active in any individual diploid genome. The L1 has also been responsible for genomic insertion of processed pseudogenes and more than a million non-autonomous retrotransposons, mainly Alus and SVAs. Together, this mass of genomic baggage has had, and continues to have, profound effects on gene organization and expression. Consequently a number of molecular mechanisms have evolved to prevent the unchecked expansion of endogenous retroelements. We demonstrate that the putative RNA helicase MOV10, recently discovered to limit production and infectivity of retroviruses, also profoundly inhibits retrotransposition of L1s, Alus, and SVAs in cell culture. Microscopy and immunoprecipitation show a close association of MOV10 protein with the L1 ribonucleoprotein particle. This study reveals a novel factor that interacts with the L1 retrotransposon to modulate its activity, and it increases our understanding of the means by which the cell coexists with these genomic “parasites.”

## Introduction

MOV10 was originally identified as a protein that prevents infection of mice by Moloney murine leukemia virus [1], [2], and has been classified as a member of the UPF1p family of SF-1 ATP-dependent RNA helicases [3]–[6]. Evidence implicates MOV10 and its relatives in small RNA regulation of gene expression. MOV10 is a homolog of SDE3, a helicase necessary for post-transcriptional gene silencing in *Arabidopsis*, armitage, a protein involved in RISC assembly and piRNA control of double-strand RNA viruses and endogenous retroelements in *Drosophila*, and ERI-6/7, which acts with Argonaute protein ERGO-1 to generate specific subsets of siRNAs in *Caenorhabditis elegans* [6]–[9]. In humans, the 1003-amino acid MOV10 (Figure S1) is present in a multi-protein complex with RISC components HIV-1 TAR RNA-binding protein (TRBP) and eukaryotic translation initiation factor 6 (eIF6). Depletion of MOV10 interferes with RNAi activity [10], [11].

MOV10 interacts with Argonaute 1 (AGO1) and AGO2 in mRNA processing (P-) bodies and stress granules (SGs), dynamic cytoplasmic aggregates that participate in mRNA decay and sequester stalled mRNA translation complexes during times of cellular stress [10]. MOV10 is also present in large complexes with the apolipoprotein B mRNA editing complex 3 members APOBEC3G and APOBEC3F, and colocalizes with these proteins in cytoplasmic granules [3], [12], [13]. Members of the APOBEC3 family of cytosine deaminases were originally defined by their ability to hypermutate reverse transcripts of human immunodeficiency virus (HIV) RNA, and subsequently were found to inhibit retrotransposition of endogenous retroelements by a poorly defined process that does not appear to involve deamination of cDNA (reviewed in refs. [14], [15]).

Several reports have demonstrated that overexpression of MOV10 severely impairs infectivity of HIV-1 and other lentiviruses. However, evidence is conflicted as to the effects of depleting endogenous MOV10, claiming that inhibiting MOV10 variously reduces HIV-1 infectivity by 50% [3], increases infectivity 3-fold [16], and reduces HIV-1 virus production 2-fold without changing infectivity [17]. Nevertheless, analogous to the activity of APOBEC3 proteins, it is reasonable to propose that MOV10 may function not only in retroviral control but also in the control of endogenous retrotransposons. Tellingly, it has recently been reported that MOV10-like-1 (MOV10L1), a protein related to MOV10 (47% identity across 466 amino acids), is expressed in mouse male germ cells where it interacts with piRNA proteins MILI and MIWI, and is involved in transcriptional silencing of L1 and long terminal repeat (LTR) retrotransposons [18]–[20].

Over two-thirds of the human genome may be repeat-derived, mostly from transposable elements [21]. Two major groups of mobile DNA exist. Class II elements or DNA transposons move by a “cut and paste” mechanism, although no currently active transposons have been identified. Class I elements move in a “copy and paste” manner involving reverse transcription of an RNA intermediate and insertion of its cDNA copy at a new site in the genome. Class I has two major subgroups. LTR retrotransposons include endogenous retroviruses (HERVs), relics of past rounds of germline infection by viruses that lost their ability to reinfect and became trapped in the genome. No active HERVs are known. Transposition of non-LTR retrotransposons is fundamentally different. RNA copies of these elements are likely carried back into the nucleus where their reverse transcription and integration occurs in a single step on the DNA itself.

LINE-1 (L1) non-LTR retrotransposons comprise at least 17% of the human genome, and are its only autonomously active mobile DNA. Up to 5% of newborn children are estimated to have a new retrotransposon insertion [22], although rates during early embryogenesis and in selected somatic cell types may be significantly higher. L1s have also been responsible for genomic insertion of thousands of human processed pseudogenes and over a million non-autonomous SINE retrotransposons, principally Alus and SVAs [23]. There are 96 known human disease-causing insertions of L1s, Alus, and SVAs [24].

Alu elements are non-autonomous retrotransposons that derive originally from a portion of the 7SL RNA component of the protein signal recognition particle (reviewed in [25]). Unlike L1s, Alus are transcribed by polymerase III, are short (about 300 bp in length), and encode no protein, and so are dependent upon the L1 retrotransposition machinery *in trans* for their insertion into the genome (reviewed in [26]). At this they have been extraordinarily successful, comprising about 10% of human DNA and exerting profound effects on genome organization and gene expression.

Hominid genomes also contain the composite retrotransposon termed SVA, an acronym for its component parts: 1) CCCTCT hexameric repeats, 2) sequence with homology to two antisense Alu fragments, 3) variable number of tandem repeats (VNTR), and 4) sequence derived from the ENV gene and right LTR of an extinct HERV-K (SINE-R). There are roughly 2700 SVA copies in the human genome, most of which are full-length (2–3 kb), and some of which are active [27]–[29]. SVA is the youngest active human retrotransposon and has been associated with seven cases of single-gene disease [24].

Here we reveal a close association of both exogenous and endogenous MOV10 with the L1 ribonucleoprotein particle (RNP). Using established cell culture reporter assays, we demonstrate that MOV10 protein, first shown to inhibit retroviruses, has an expanded repertoire that includes suppression of non-LTR retrotransposition. Some phylogenetic analyses suggest that non-LTR retrotransposons are the likely progenitors of retroviruses and LTR retrotransposons [30]. This raises the question of whether MOV10, co-opted by the cell to reduce the threat of invading retroviruses, may have originally evolved to suppress the activity of an enemy within.

## Results

### MOV10 associates with the L1 RNP

L1 expresses a 6-kb bicistronic RNA that encodes a 40 kD RNA-binding protein (ORF1p) of essential but uncertain function for retrotransposition, and a 150 kD ORF2 protein with endonuclease and reverse transcriptase (RT) activities. Epitope-tagging of either of these proteins permits the immunoprecipitation (IP) of L1 RNP particles from cells, along with associated cellular proteins [31], [32].

**Figure 1:**
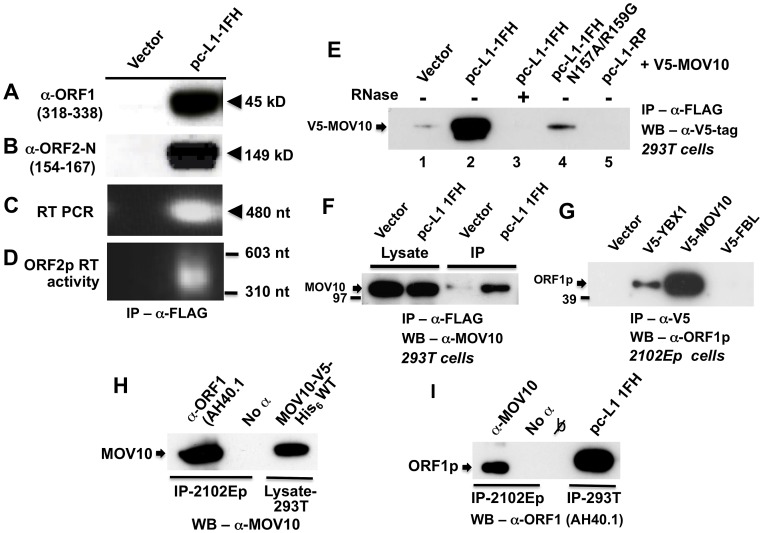
Construct pc-L1-1FH successfully immunoprecipitates basal L1 RNP complexes (ORF1p, ORF2p, and L1 RNA) from 293T cell lysates following α-FLAG purification. Detection in purified immunoprecipitates of (A) FLAG-HA-tagged ORF1 protein, (B) ORF2 protein, (C) L1 RNA detected by RT-PCR, and (D) ORF2 reverse transcriptase activity, assayed as described by Kulpa et al. [35]. (E–I) Endogenous and ectopically expressed MOV10 protein and L1 ORF1p associate in multiple cell lines. (E) Immunoprecipitation of V5-tagged MOV10 by FLAG-tagged pc-L1-1FH depends upon the presence of RNA (lanes 2 and 3). A double point mutation in ORF1 of pc-L1-1FH known to inhibit RNA-binding, prevents efficient co-IP of MOV10 protein (lane 4). Removing the FLAG-HA-tag from pc-L1-FH (pc-L1-RP) prevents IP of the L1 RNP and MOV10 protein on α-FLAG agarose (lane 5). (F) pc-L1-1FH-generated RNPs associate with endogenous MOV10 protein in 293T cells. (G) Transfected V5-tagged MOV10 and cold shock domain protein YBX1, but not fibrillarin (FBL) or empty vector, immunoprecipitate endogenous ORF1p from 2102Ep cells. (H) α-ORF1 (AH40.1) antibody co-IPs endogenous MOV10 protein from 2102Ep cells (lane 1). Lane 3: lysate of 293T cells transfected with MOV10-V5-His_6_ WT as a marker for MOV10 protein. (I) Similarly, immunoprecipitation using α-MOV10 antibody yields endogenous ORF1p (lane 1). Lane 4: α-FLAG-tag IP from 293T cells transfected with pc-L1-1FH as a marker for ORF1p.

We tagged the C-terminus of ORF1 in L1-RP (an L1 highly active in cell culture assays; [33]) with a tandem hemagglutinin (HA)-FLAG tag to create the construct pc-L1-1FH. Tagging ORF1 in this manner diminishes but maintains activity of L1-RP in an enhanced green fluorescent protein (EGFP)-reporter assay for cell culture retrotransposition [34] (Figure S2). All evidence indicates that pc-L1-1FH is capable of immunoprecipitating basal L1 RNP complexes from cell lysates. Following transfection in 293T cells and α-FLAG agarose purification, we detected in cytoplasmic immunoprecipates both ORF1 and ORF2 proteins (Figure 1A and 1B), L1 RNA (Figure 1C), and robust RT activity (Figure 1D) as determined by an *in vitro* PCR-based assay [35].

To identify cellular proteins that associate with the L1 RNP, we subjected pc-L1-1FH and pcDNA6 empty vector control IP samples recovered from 293T cells to mass spectrometry (MS) analyses. Among approximately 100 non-ribosomal proteins unique to the pc-L1-1FH sample and identified by at least two predicted peptides (paper in preparation), we detected Moloney leukemia virus 10, homolog (mouse), or MOV10 (Table S1).

We cloned MOV10 cDNA with N-terminal V5-epitope tag and showed by Western blotting that its protein could co-IP with tagged pc-L1-IFH RNPs after transfection in 293T cells. This association was RNA-dependent and was lost upon treatment with RNase (Figure 1E, lanes 2 and 3). Altering two conserved residues in ORF1p (N157A/R159A), a mutation that diminishes RNA-binding [36], greatly attenuated immunoprecipitation of MOV10 (Figure 1E, lane 4). L1-RP lacking ORF1 tag failed to IP MOV10 (Figure 1E, lane 5).

FLAG-tagged pc-L1-1FH was also able to pull-down endogenous MOV10 protein from 293T cells (Figure 1F). We next tested the ability of V5-MOV10 to co-IP endogenous ORF1 protein from 2102Ep cells, a human embryonal carcinoma line that expresses L1 ORF1p at particularly high levels [31]. Using the well-characterized α-ORF1p AH40.1 polyclonal antibody [37], we detected endogenous ORF1p in association with V5-MOV10 and V5-YBX1, the latter an RNA-binding protein previously reported to bind with the L1 RNP [31], but not V5-tagged fibrillarin (FBL) or empty vector (Figure 1G). Finally, we determined that endogenous ORF1p co-IPs with endogenous MOV10 protein from 2102Ep cells (Figure 1H and 1I).

Therefore, both exogenously expressed and endogenous MOV10 proteins associate with the L1 RNP and ORF1p in multiple cell lines.

### Colocalization of MOV10 in cytoplasmic granules with ORF1p

ORF1p is a predominantly cytoplasmic protein, but can be detected in nuclei and enters nucleoli of a minor percentage of cells [38]. Previously, we reported that endogenous or ectopically-expressed ORF1p, in the absence of external stress applied to the cell, itself nucleates the formation of stress granules and colocalizes with their markers [31], [39]. SGs are cytoplasmic aggregates induced by a range of stresses and contain stalled pre-initiation mRNA complexes [40]. When expressed from a full-length L1 construct, ORF1p is present in SGs as an RNP together with L1 RNA and ORF2p [32], [39]. In stressed cells, ORF1p also colocalizes with Ago2 and human fragile X protein (FMRP), components of the RNA-induced silencing complex RISC [31]. ORF1p cytoplasmic granules typically do not colocalize, but not infrequently juxtapose with processing bodies (PBs), proposed sites of mRNA turnover and decay [31], [41]. PBs and SGs are often found in close proximity and can exchange components [40].

**Figure 2:**
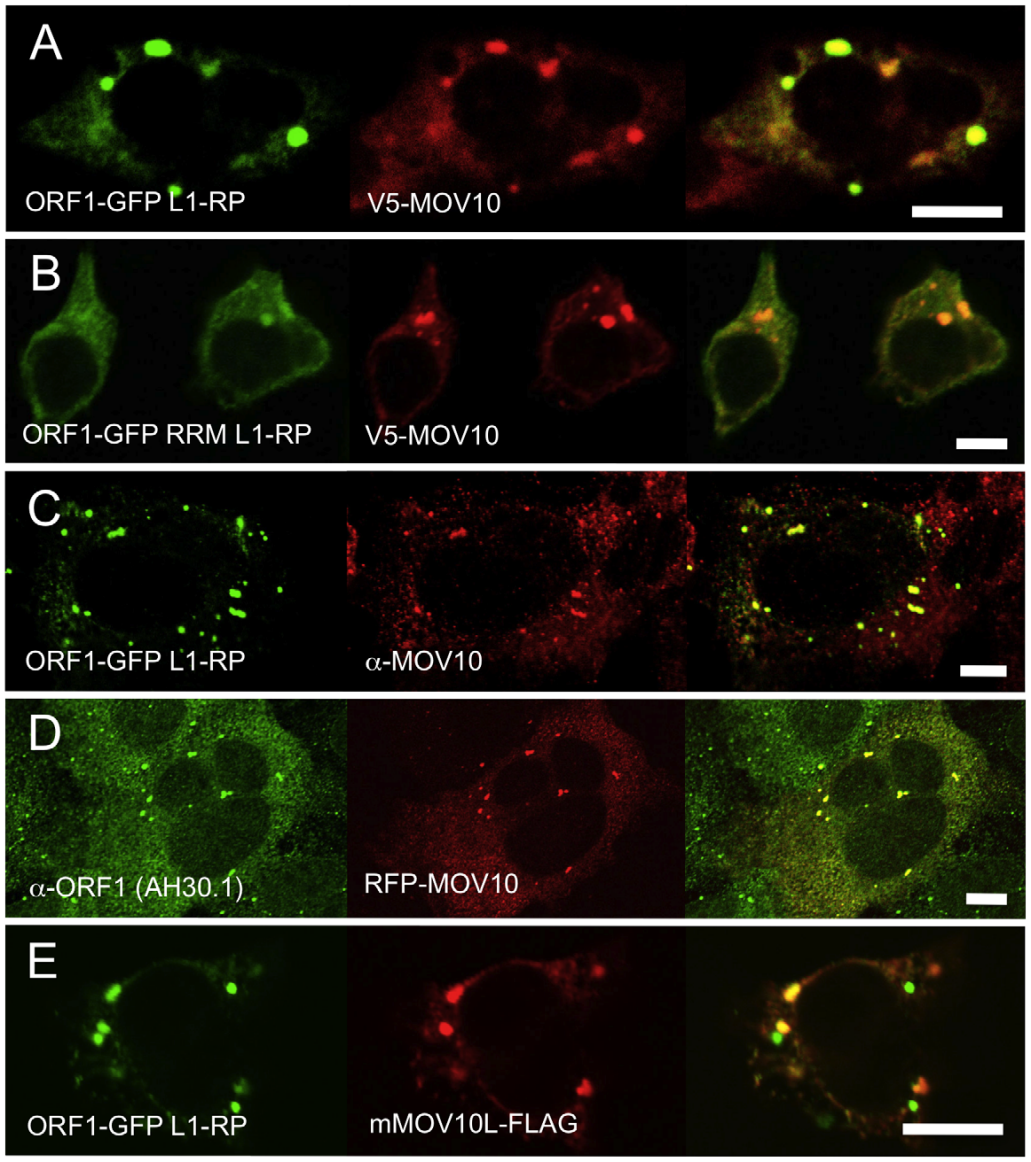
Ectopically expressed and endogenous MOV10 closely colocalizes with L1 ORF1p in multiple cell lines. (A) EGFP-tagged ORF1p colocalizes with V5-tagged MOV10 in the cytoplasm and foci of 293T cells. (B) A mutation in the RRM RNA-binding domain of EGFP-ORF1p diminishes, but does not abolish, colocalization with MOV10 in cytoplasmic foci. (C) EGFP-ORF1p colocalizes with endogenous MOV10 in cytoplasmic granules of 2102Ep cells. (D) Endogenous ORF1p extensively colocalizes with RFP-tagged MOV10 in 2102Ep cells. (E) In 293T cells mouse mMOV10L is present in some, but not all ORF1p-marked cytoplasmic bodies, and significantly less often than MOV10. Scale bar: 10 µm.

Meister et al. [10] demonstrated colocalization of MOV10 with AGO1 and AGO2, proteins that concentrate in PBs together with miRNAs and other components of the RNAi pathway [42]–[44]. Furthermore, Gallois-Montbrun et al. [13] reported that APOBEC3G and associated MOV10 colocalize in PBs of unstressed and in SGs of stressed cells. Contrarily, El Messaoudi-Aubert et al. [45] reported endogeous MOV10 to be mostly nuclear. In agreement with Sim et al. [46], we found MOV10 protein in unstressed cells to be predominantly cytoplasmic, mirroring the distribution of L1 ORF1p (Figure 2A and 2D).

To assess MOV10 association with the L1 RNP, we cotransfected V5-tagged MOV10 together with ORF1-GFP L1-RP, a construct containing a CMV promoter, ORF1 C-terminally tagged with EGFP, and intact downstream L1 sequence [39]. There was a close coincidence of MOV10 and ORF1p in cytoplasmic granules (Figure 2A). Notably, in 293T cells granules appeared larger and ORF1p signal diminished from that observed when ORF1-EGFP fusion protein was expressed in the absence of MOV10.

We previously reported [31] that ORF1-GFP L1-RP having point mutations near the N-terminus of a subsequently described ORF1p RRM RNA binding domain, fails to form cytoplasmic foci in cultured cells. One critical change is R159A, which has been shown to inhibit RNA-binding by ORF1p [36]. Although ORF1-GFP RRM L1-RP, a construct with RRM altered in this region, cannot induce cytoplasmic foci when expressed alone, when coexpressed with MOV10, foci formation is attenuated but readily detectable (Figure 2B). Since the R159A mutation does not completely abolish ORF1p RNA-binding [36], ORF1-GFP RRM L1-RP RNPs retain some ability to interact with MOV10 (also compare Figure 1E, lanes 2 and 4). Together, these data suggest that MOV10 protein can recruit ORF1p to stress granules through an RNA intermediate.

We next assayed for colocalization of endogenous ORF1p and MOV10 proteins. ORF1p-EGFP overlaps endogenous MOV10 protein in almost all visible cytoplasmic granules (Figure 2C). Conversely, colocalization of red fluorescent protein (RFP)-tagged MOV10 with endogenous ORF1p was seen in most foci of 2102Ep cells (Figure 2D).

Finally, we examined 293T cells for association of the L1 RNP and FLAG-tagged mouse MOV10L, the MOV10 testis-specific paralog [20]. Like human MOV10, mMOV10L forms prominent cytoplasmic foci, although their direct overlap with ORF1p granules is less striking than for MOV10. Many mMOV10L and ORF1p foci do not overlap, while others appear more contiguous than coincident (Figure 2E). Perhaps MOV10L aligns more closely with L1 RNPs in male germ cells where it is specifically expressed.

To summarize, both exogenous and endogenous ORF1p and MOV10 proteins are directed to the same cytoplasmic compartments.

### MOV10 inhibits retrotransposition in a cell culture assay

**Figure 3:**
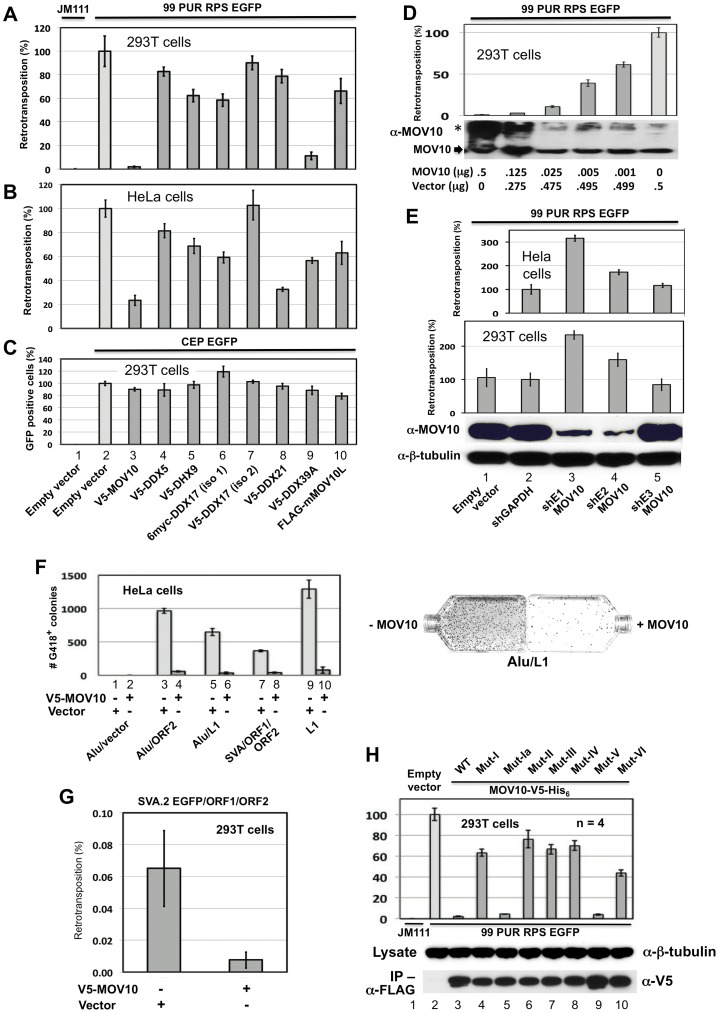
Evidence from cell culture retrotransposition assays that MOV10 inhibits insertion of non-LTR retrotransposons. (A) The reporter construct 99 PUR RPS EGFP was cotransfected in 293T cells with empty vector (pcDNA3) or constructs expressing V5-MOV10 or other epitope-tagged L1 RNP-associated helicases. Constructs are numbered and named at the bottom of Panel C. Five days later, percentages of EGFP-positive cells (ie. cells with a retrotransposition event) were determined by flow cytometry. Each construct pair was tested in quadruplicate (n = 4), and results are normalized to pcDNA3 empty vector control (#2). An L1 mutant, 99 PUR JM111 EGFP (JM111), was used as negative control for retrotransposition and FACs gating (#1). V5-DDX17 (isoform (iso)2, #7), is generated from an abbreviated transcript variant spanning only residues 549 to 729 of the C-terminal region of full-length DDX17 (#6). (B) Same as (A) in HeLa-HA cells. (C) To assess transfection efficiency and cell toxicity, helicase constructs were cotransfected with CEP-EGFP and fluorescent cells were assayed 4 days later. Results are from 293T cells. (D) Expression of MOV10 inhibits retrotransposition in a dose-dependent manner. Decreasing microgram amounts of MOV10-expessing plasmid, mixed with empty vector to normalize DNA concentrations, were cotransfected with the L1-reporter construct, 99 PUR RPS EGFP, and assayed for retrotransposition at 5 days. Western blot analysis of cytoplasmic lysates (below) shows that even very small amounts of exogenous MOV10 protein can inhibit retrotransposition. High-molecular weight aggregates of overexpressed MOV10 protein are also visible on overexposed gels (marked by *). (E) Loss of endogenous MOV10 expression increases retrotransposition. Stable Hela and 293T cell lines were established by infection with lentiviral particles expressing shRNAs against MOV10, GAPDH or empty vector, followed by selection with puromycin. These cell lines were then tested for retrotransposition competency of 99 PUR RPS EGFP. Results are normalized to empty vector control. Bottom panels: Western blot showing that shE1 and shE2 decrease endogenous MOV10 protein levels by about 90 percent in 293T cells, and loading control blot showing β-tubulin. (F) Overexpression of MOV10 decreases Alu and SVA retrotransposition in HeLa-HA cells. As described in Dewannieux et al. [49], a Ya5 Alu is cloned in a plasmid containing the 7SL pol III enhancer and neo^TET^ cassette interrupted by a Tetrahymena self-splicing intron. Upon transcription, the intron is spliced out. When this construct is co-expressed with L1 ORF2 alone (#3 and 4) or full-length L1-RP (#5 and 6), Alu RNAs are reverse transcribed along with the *neo* gene and integrated into the genome to confer neomycin resistance. Following 15 days of treatment with neomycin, resistant colonies were stained and counted. Either empty vector (#1, 3, 5, 7, and 9) or V5-MOV10 plasmid (#2, 4, 6, 8 and 10) was included in the reactions. Retrotransposition data for SVA.2 and L1-RP containing the *mneoI* antibiotic-selection cassette [47] are also shown (#7–10). Colony counts are not normalized. To the right are representative T_75_ flasks with Giemsa-stained Alu retrotransposition-positive colonies in the absence (left) or presence (right) of MOV10. (G) The SVA EGFP retrotransposition assay [28] was performed in 293T cells with SVA.2 EGFP cotransfected with constructs expressing L1 ORF1 and ORF2 separately, in the presence or absence of MOV10. (H) Mutations in helicase motifs I–VI impede anti-retrotransposition activity of MOV10. Top panel: MOV10-V5-His_6_ wild-type and mutant proteins [51] were tested for effect on retrotransposition of 99 PUR RPS EGFP. Results are normalized to empty vector control (#2). Middle panel: mutant and wild-type MOV10-V5-His_6_ proteins are expressed at similar levels in 293T cells. Bottom panel: mutant and wild-type MOV10-V5-HIS_6_ proteins all efficiently co-IP with L1 RNPs expressed from pc-L1-1FH.

Cognizant of the ability of MOV10 to inhibit retroviral infection, and of the related MOV10L protein to suppress transcription of L1s and IAP retrotransposons in male germ cells of knock-out (KO) mice [20], we wished to determine if MOV10 inhibits genomic insertion of retrotransposons in cells. For this purpose, we employed widely-used cell culture L1 retrotransposition assays originally developed in our lab [34], [47]. An EGFP reporter cassette, interrupted by an intron in opposite transcriptional orientation and inserted into the 3′ UTR of L1-RP (construct 99 PUR RPS EGFP), is expressed only when the L1 transcript is spliced, reverse transcribed, its cDNA inserted in the genome, and the EGFP reporter gene expressed from its own SV40 promoter. We cotransfected construct 99 PUR RPS EGFP [34], together with empty (pcDNA3) or tagged MOV10 vectors, in 293T and HeLa cells, and 5 days post-transfection assayed for fluorescent cells by flow cytometry. Coexpressed MOV10 caused a precipitous decrease, over 95%, in the number of retrotransposition-positive 293T cells relative to empty vector control (Figure 3A, compare bar #2 and 3). In HeLa cells, MOV10 also caused a significant, but less dramatic, decline in retrotransposition when assayed by EGFP reporter (80%; Figure 3B, #2 and 3). However, by using the sensitive *mneoI*-reporter assay [47], a more pronounced loss of retrotransposition (95%) was seen in HeLa cells (Figure 3F, #9 and 10). Ectopically-expressed MOV10 inhibits L1 retrotransposition in a dose-dependent manner, and its effects remain significant at protein levels undetectable by Western blotting (Figure 3D).

The duration of the cell culture retrotransposition assay is necessarily long: in 293T cells the first retrotransposition events appear after 24 hours, and slowly accumulate in number over the course of the experiment. Therefore, it is important to determine if coexpressed proteins cause toxicity that might bias interpretation of results. Both to determine transfection efficiency and to assay for potential MOV10-related toxicity, we transfected pCEP-EGFP, a vector that constitutively expresses EGFP, together with empty or V5-tagged MOV10, and after 4 days performed flow cytometry. Loss of fluorescence due to MOV10 cotransfection was minimal, and could not account for the severe reduction in levels of retrotransposition (Figure 3C, #2 and 3). Overexpression of MOV10 also showed little cytotoxicity as determined by trypan blue staining at 4 days (not shown).

We next asked whether endogenous MOV10 inhibits L1 retrotransposition. Lentiviral particles derived from three different shRNA constructs against human MOV10 were used to generate stable HeLa and 293T cell lines. Two of the shRNA constructs (shE1 and shE2) silenced MOV10 protein expression by 90%, while a third (shE3) had no apparent effect (Figure 3E, third panel. lanes 3–5). Depletion of endogenous MOV10 by the shE1 and shE2 constructs enhanced L1 retrotransposition up to 3-fold, as compared with cells infected with viral particles generated from empty vector, shE3, or an shRNA against GAPDH (Figure 3E, first and second panels). We predict that complete inhibition of MOV10 protein would permit even higher levels of retrotransposition. As shown in Figure 3D, even low levels of exogenous MOV10 cause significant loss of L1 activity. The potential upper limit of retrotransposition is unknown, however: a number of mechanisms apart from MOV10 operate in cells to tamp down endogenous retrotransposition (reviewed in [48]).

We also employed the assay of Dewannieux et al. [49] to ascertain the effects of MOV10 overexpression on Alu retrotransposition. In the presence or absence of MOV10, Alu-neo^Tet^ was cotransfected with either empty vector or a retrotransposition “driver” plasmid: i.e. pCEP 5′UTR ORF2 No Neo (containing L1 5′UTR, ORF2 and CMV promoter [50]) or pcDNA6 L1-RP (L1-RP lacking a reporter cassette). HeLa cells were expanded to T_75_ flasks at 18 hours post-transfection, and selected on neomycin from day 5 to day 20, after which time colonies (each representing a unique retrotransposition event) were stained and counted. Alu-neo^Tet^ with empty vector generated almost no colonies, but in the presence of a driver plasmid numerous neomycin-resistant foci were detected. Introducing MOV10 reduced foci number by almost 95 percent (Figure 3F, #3–6).

Cell-culture assays for SVA retrotransposition have recently been described [28], [29]. A canonical SVA element, SVA.2, tagged with *mneoI* indicator cassette, was cotransfected in 293T cells together with two driver plasmids expressing L1 ORF1 and L1 ORF2 separately, and either empty vector or V5-MOV10. Following expansion into T_75_ flasks and selection with neomycin as described above, the presence of MOV10 protein was found to restrict SVA retrotransposition by about 90 percent (Figure 3F, #9 and 10). This result was confirmed in 293T cells using EGFP-reporter tagged SVA.2 [28] and FACs analysis of retrotransposition (Figure 3G).

Thus, MOV10 protects the genome from insertional mutagenesis by all active human retrotransposons.

### Efficient suppression of L1 retrotransposition requires intact helicase motifs

MOV10 shares with other helicases seven conserved motifs in its C-terminus (motifs I, Ia, II, III, IV, V, and VI; Figure S1). We obtained from the Zheng lab (Michigan State University) [16] seven mutant clones each with alanine substitution(s) of critical residues within the helicase motifs, and tested these clones, along with their wild-type parent construct (MOV10-V5-His_6_ WT), for effect on L1 retrotransposition in cell culture. Mutations in all motifs except Ia and V (Figure 3H, top panel, #5 and 9) suppressed the ability of MOV10 to restrict L1 retrotransposition. Consistent with our findings, Abudu et al. [51] reported that all mutations except Mut V suppressed anti-HIV-1 activity of MOV10. Mut Ia contains a single altered residue: perhaps this is not sufficient to inactivate the domain and alter retrotransposition.

All MOV10 mutants except Mut V fail to enter HIV virions [51]. This does not seem to be the case with the L1 RNP; wild-type (MOV10-V5-His_6_) and its mutant proteins were expressed at similar levels in 293T cells, and all co-IPed efficiently with pc-L1-1FH (Figure 3H, middle, bottom panels). The anti-retrotransposon role of these motifs is therefore unclear. Motifs I and II are Walker A and B boxes, respectively, and may catalyze hydrolysis of purine nucleoside triphosphate to provide energy for helicase activity, while the other five motifs may contribute to RNA or DNA binding [52].

We conclude that intact MOV10 helicase motifs, and possibly helicase activity, are critical for efficient suppression of retrotransposition.

### Inhibition of retrotransposition is not a general feature of ATP-dependent RNA helicases

We next wished to determine if other RNA helicases might also inhibit L1 retrotransposition in a manner similar to MOV10 protein. In addition to MOV10, we detected by IP and MS-sequencing additional ATP-dependent RNA helicases associated with the L1 RNP (Table S1), including DEAD box polypeptide 5 (DDX5/p68), its paralog DDX17 (p72), and DEAH-box helicase DHX9 (ATP-dependent RNA helicase A, RHA). Like MOV10, all three of these helicases have been detected within the RNAi pathway. DHX9 binds with RISC and appears to be involved in the loading of guide-strand siRNAs [53]. DDX5 and DDX17 are components of the large hDROSHA-processing complex and may facilitate pri-miRNA, as well as pre-rRNA processing and ribosome biogenesis [54]–[56]. Also identified by our MS analyses were DDX21 (RNA helicase II/Guα), a nucleolar DEHD/X-box helicase involved in rRNA processing and JUN-activated transcription [57], [58], and DDX39A, a close homolog of UAP56 which plays a role in spliceosome assembly and nuclear export of spliced and unspliced mRNAs [59].

**Figure 4:**
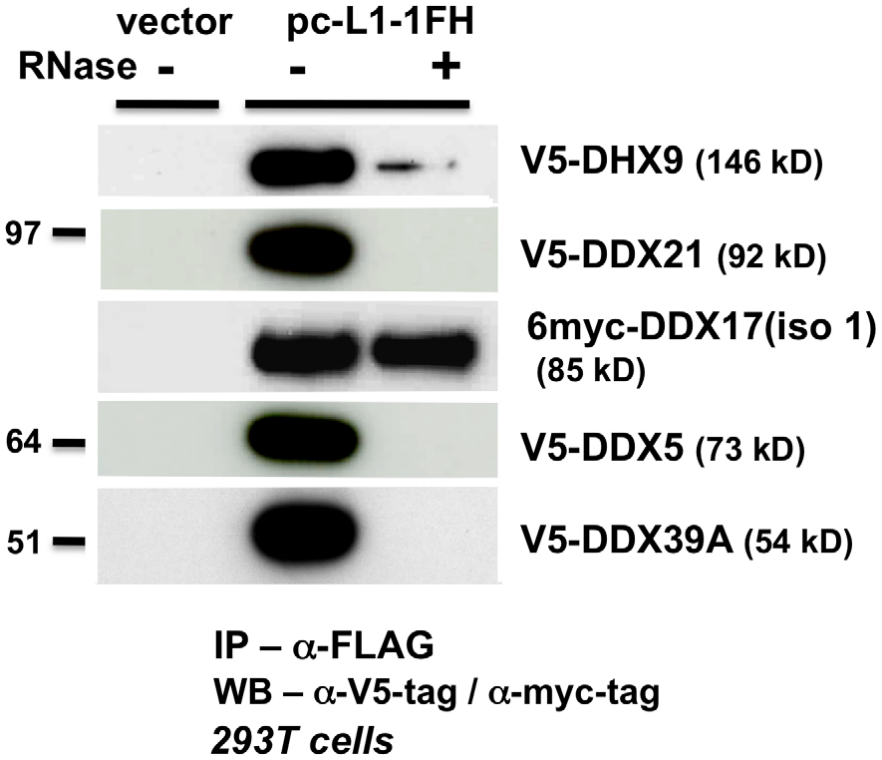
ATP-dependent RNA helicases other than MOV10 also associate with L1 RNPs expressed from pc-L1-1FH. In α-FLAG immunoprecipitates, only association with myc-tagged full-length DDX17 (iso1) is resistant to RNase digestion.

Like MOV10, V5 epitope-tagged DDX5, DHX9, DDX21, and DDX39A proteins co-IPed with tagged L1 RNP complexes in an RNA-dependent manner (Figure 4). Only myc-tagged DDX17 resisted RNase treatment, predicting a tight association with the L1 RNP or direct binding to ORF1p. We failed in attempts to coimmunoprecipitate FLAG-tagged mouse MOV10L protein together with L1-RP tagged with a tandem affinity purification (TAP) tag on ORF1p (not shown).

As described above for MOV10, we expressed tagged DDX5, DHX9, DDX21, DDX17, DDX39A, and mMOV10L, together with construct 99 PUR RPS EGFP and assayed for altered rates of retrotransposition in 293T and HeLa cell culture (Figure 3A and 3B, #4–10). For the most part, ectopic expression of these helicases only modestly decreased retrotransposition. DDX39A lowered L1 retrotransposition 90% in 293T cells, but not in HeLa cells (#9). The converse was true for DDX21 (#8). mMOV10L did not greatly alter L1 retrotransposition in cell culture (#10): this was unexpected, since this protein strongly suppresses L1 and IAP expression in mouse spermatocytes [20]. However, mMOV10L interacts with piRNA proteins MILI and MIWI, and so may function in retrotransposon control only in germ cells. None of these tagged helicase constructs showed significant toxicity or differences in transfection efficiency in cells (Figure 3C, #4–10).

Thus, although several ATP-dependent RNA helicases associate with the L1 RNP, only MOV10 strongly inhibits retrotransposition in multiple cell lines.

### MOV10 suppresses L1 expression and decreases cytoplasmic L1RNPs

**Figure 5:**
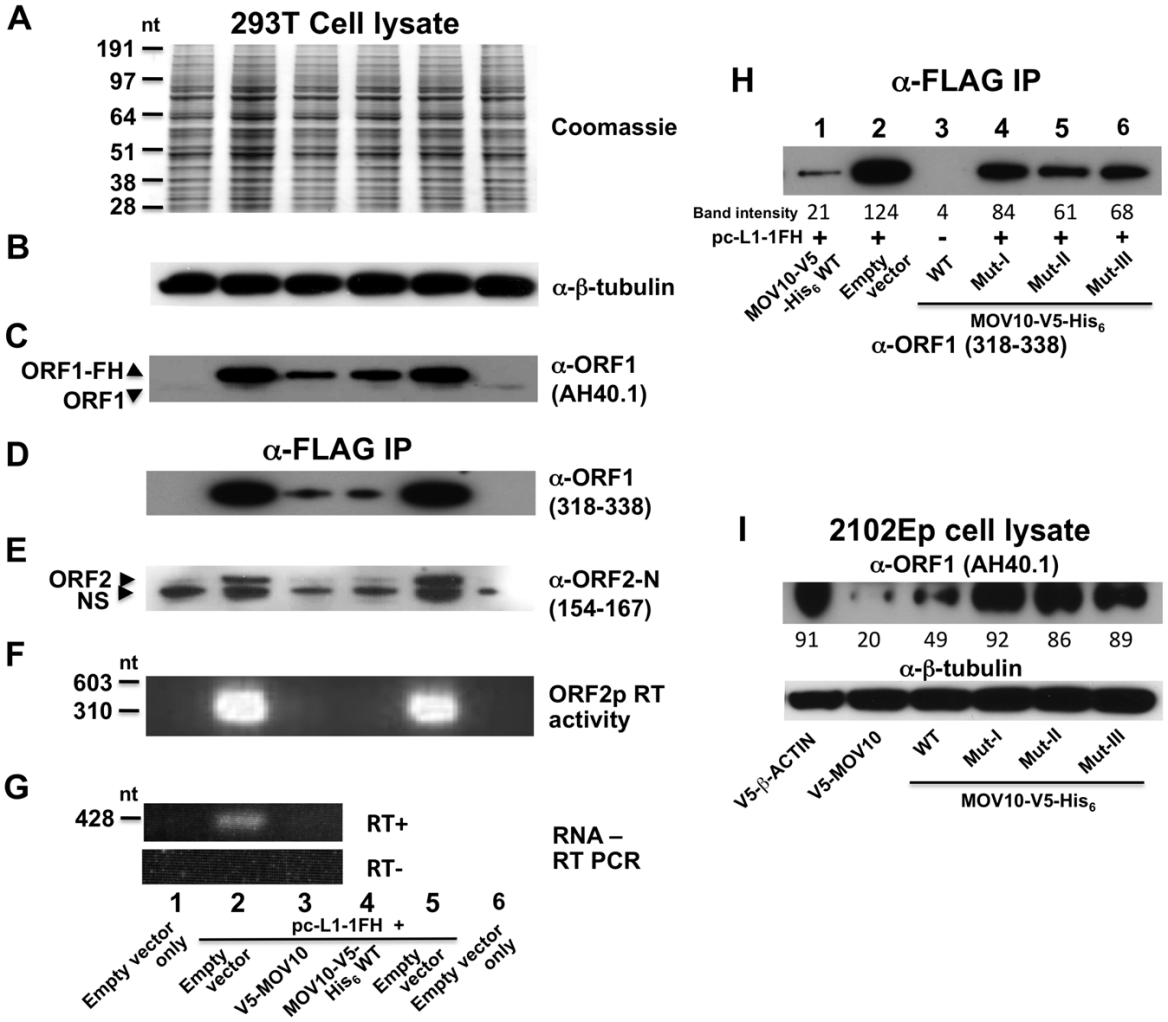
MOV10 inhibits exogenous L1 RNP expression in cells. (A–C) Analysis of 293T cell lysates showing that MOV10 expression has no discernable effect on total protein production as determined by (A) Coomassie blue staining, or (B) Western blot detection of constitutively expressed proteins. (C) However, MOV10 attenuates expression of ORF1p from pc-L1-1FH. Note, α-ORF1 AH40.1 only faintly detects endogenous ORF1 protein in this blot exposure. (D–G) Analysis of immunoprecipitate samples following IP of pc-L1-1FH. Levels of (D) ORF1p, (E) ORF2p, (F) ORF2p RT activity, and (G) L1 RNA are strongly diminished in the presence of MOV10. α-ORF2-N occasionally detects a slightly smaller than expected band in untransfected cells (labeled NS in (E)). It is not known if this band is non-specific or a truncated form of endogenous ORF2p. Lane names and numbers at the bottom refer to all panels A–G. (H) Analysis of 293T immunoprecipitate samples following α-FLAG IP of cotransfected pc-L1-1FH. Introducing mutations in helicase domains of MOV10 significantly abrogates its inhibition of ORF1p expression from pc-L1-1FH (compare also with panel D). (I) V5-MOV10 protein was expressed in 2102Ep cells, and endogenous ORF1 protein was detected in lysates with α-ORF1 AH40.1 antibody. Endogenous ORF1p levels decrease in the presence of MOV10 wild-type protein, but to lesser degree with MOV10 mutants (lower panel, lanes 4–6). ImageJ software (NIH) was used to quantitate band intensities, and their absolute readings are arrayed below the figure panels (H and I).

Finally, we examined the effect of MOV10 protein on expression of L1s in cell culture. Ectopic expression of MOV10 was without obvious effect on global protein expression, as evidenced by Coomassie blue staining of cell lysates and Western blot detection of constitutively expressed proteins such as β-tubulin (Figure 5A and 5B). On the other hand, levels of ORF1p and ORF2p expressed from pc-L1-1FH were significantly reduced in both cell lysates and immunoprecipitates in the presence of transfected MOV10 (Figure 5C–5E). ORF2 RT activity was almost undetectable, and L1-RP RNA levels were diminished (Figure 5F and 5G). Introducing mutations in MOV10 helicase domains I, II, or III significantly restored expression of ectopic ORF1p as compared with inhibition by wild-type MOV10, consistent with the inability of these mutants to strongly restrict retrotransposition (Figure 5H, compare lane 1 with lanes 4–6; Figure 3H).

Over-expression of wild-type MOV10 protein also caused a modest reduction of endogenous ORF1 protein levels in 2102Ep cells, and this suppression was relieved by mutations in MOV10 (Figure 5I, upper panel). This is analogous to observations that cellular HIV gag levels decrease in the presence of increasing amounts of exogenous MOV10 [17]. Thus, MOV10 affects LINE1 expression, although it remains unclear if the effect is on transcription or post-transcriptional.

**Figure 6:**
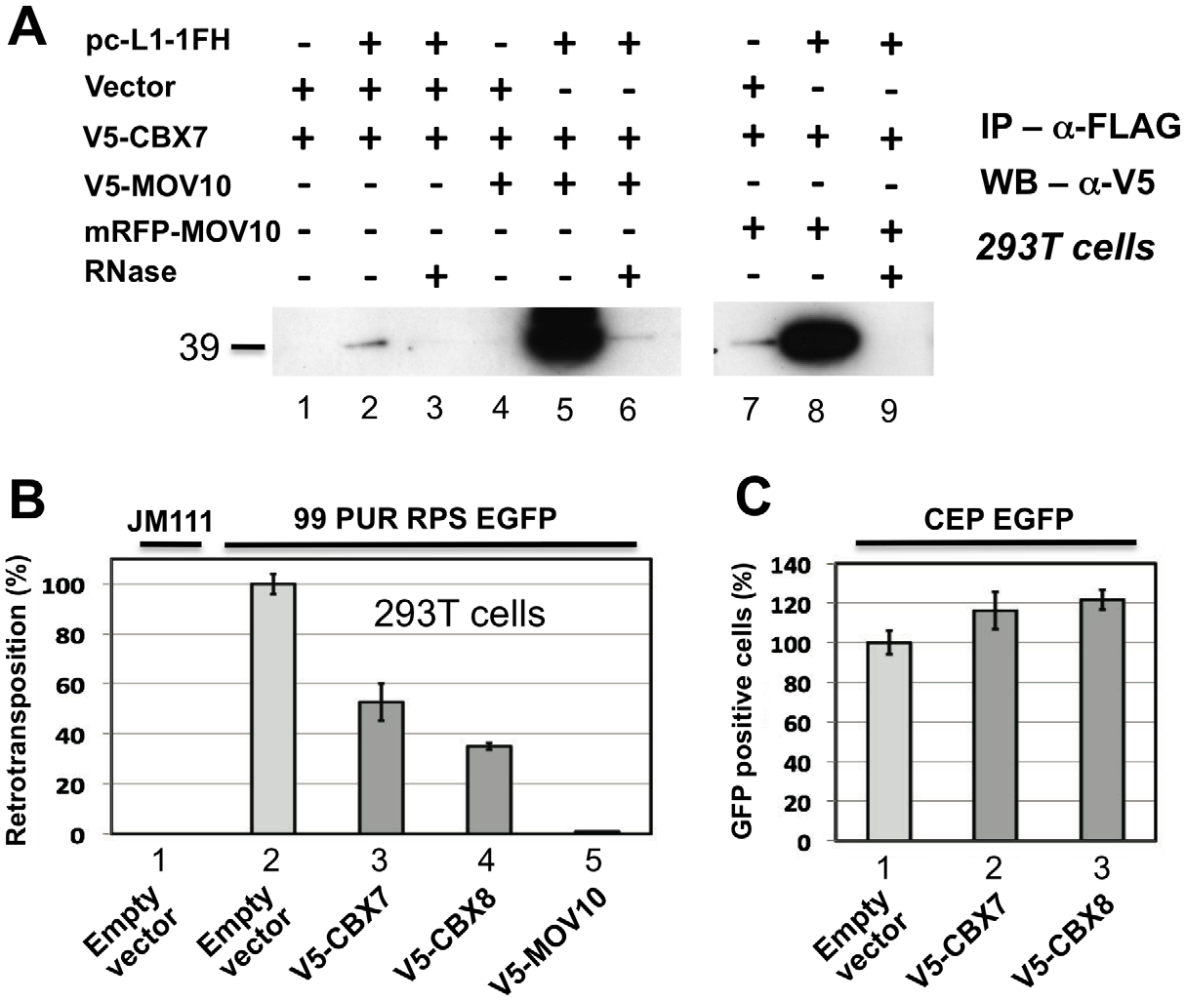
Polycomb group (PcG) multiprotein PRC1-like complex component Chromobox homolog 7 (CBX7) associates with the L1-RNP and inhibits retrotransposition. (A) Lanes 1–3: CBX7 binds weakly with the L1 RNP in an RNA-dependent manner. Lanes 4–9: Co-immunoprecipitation of V5-tagged CBX7 by pc-L1-1FH is greatly enhanced by coexpression of tagged MOV10 proteins. (B) When overexpressed in the cell culture assay, both CBX7 and PRC1 component CBX8 significantly inhibit L1 retrotransposition, without obvious cell toxicity (C).

In addition to associating with RISC, MOV10 also binds chromobox family protein CBX7 and, to lesser degree, CBX6 and CBX8, components of the Polycomb repressive complex 1 (PRC1), which is involved in maintaining some genes in a transcriptionally repressed state during development. El Messaoudi-Aubert [60] determined that shRNA-mediated knockdown of MOV10 causes up-regulation of INK4a, a known PRC1 target, accompanied by displacement of PRC1 components, histone modification, and chromatin remodeling at the INK4a promoter. We sought to determine by co-IP if Polycomb group proteins also associate with the L1 RNP. Weakly detected in the absence of MOV10, CBX7 was strongly recruited to the L1 RNP when coexpressed with V5- or RFP-tagged MOV10 (Figure 6A; CBX8 bound non-specifically to α-FLAG-agarose making IP results inconclusive; data not shown). CBX7 and CBX8 inhibit cell culture retrotransposition 50 and 60 percent, respectively (Figure 6B), in the absence of overt cellular toxicity (Figure 6C).

In addition to its role in histone modification, CBX7 recruits DNA methyltransferases (DNMTs) to chromatin [61]. MOV10 is a component of RISC, and one mechanism of small RNA gene silencing is induction of DNA methylation [62]. Therefore, wondering if MOV10 might suppress L1s by altering methylation, we queried the methylation status of the CpG island contained within the 5′ UTR of endogenous members of the young and active L1PA1 subfamily in the presence or absence of overexpressed MOV10. No differences in the methylation status of 20 CpG residues were detected by bisulfite conversion analysis (Figure S3). We also tested both sense and anti-sense promoters of the L1 5′UTR in the luciferase assay described by Yang and Kazazian [63], but detected no L1-specific change in promoter expression in the presence of exogenous MOV10 (data not shown).

How CBX7 or CBX8 might inhibit retrotransposition is unclear. Perhaps recruitment of PcG proteins to nascent L1 transcripts induces transcriptional silencing. Alternatively, association of these proteins with the L1 RNP might alter chromatin structure at sites of genome insertion, and so interfere with resolution of the L1 integrant. Chromatin immunoprecipitation (CHIP) and qPCR analyses of multiple L1 loci may determine if MOV10, together with PRC1 proteins, are recruited to L1s to suppress expression. Little information exists concerning the effects of chromatin structure on mammalian retrotransposon expression, although two studies have proposed roles for HDAC1 and HDAC2 [64], [65].

In summary, MOV10 inhibits L1 expression and the number of its cellular RNPs available for retrotransposition through an as yet undetermined mechanism. However, possible mechanisms include MOV10-mediated 1) sequestration and silencing of L1 RNPs in stress granules, 2) RISC inhibition of L1 transcription or translation, and 3) PcG protein recruitment for suppression of L1 expression.

## Discussion

Here we demonstrate that the putative RNA helicase MOV10 is a potent inhibitor of LINE1-mediated retrotransposition when overexpressed in cells. Conversely, knockdown of endogenous MOV10 results in a significant increase in levels of L1 retrotransposition. These results parallel the recently described capacity of MOV10 to diminish viral production and infectivity of HIV and other pathogenic retroviruses [3], [16], [17]. Association with the L1 retrotransposition machinery is confirmed by markedly close colocalization of MOV10 protein with ORF1p in cytoplasmic granules, and by the detection of MOV10 in RNP particles retrieved by immunoprecipitation of tagged L1 constructs. The loss of RNP association upon RNase treatment suggests that MOV10 may bind the L1 RNA, as it is also known to associate with HIV RNA and to interact with Gag in an RNA-dependent manner [16], [17]. Affinity of MOV10 protein for the L1 RNP likely facilitates the inhibition of L1 expression that we observe in cell culture (Figure 5).

The mechanism by which MOV10 inhibits retrotransposition remains unclear, but in light of MOV10 association with RISC, it is reasonable to consider that RNAi silencing is involved. Two studies have reported that L1-derived small (sm) RNAs participate in its inhibition [63], [66], but failed to verify these as genuine siRNAs. The question of siRNA control of retrotransposition therefore remains open. Stronger evidence exists that components of the germline piRNA pathway mediate retrotransposon control. Significantly, the MOV10 paralog MOV10L binds piRNA-associated proteins MILI and MIWI, and MOV10L loss in testes of knockout mice is marked by an increase in IAP and L1 expression [20].

piRNAs appear to inhibit retrotransposons by stimulating *de novo* methylation of their regulatory sequences [67]. Loss of MILI, MIWI2, or GASZ impairs IAP and L1 promoter methylation, together with a reduction in repeat-associated piRNAs and derepression of retrotransposon transcription in germ cells of male newborn mice [68]–[70]. Also, it was recently demonstrated in KO mice that loss of MVH (DDX4), an ATP-dependent RNA helicase, causes the same germ line abnormalities as loss of MILI and MIWI2, and similarly plays an essential role in *de novo* methylation and silencing of retrotransposons [71]. MVH is the homolog of Vasa, a Drosophila protein involved in piRNA production. It should be noted, however, that none of these KO mouse studies demonstrated that an increase in retrotransposon expression actually led to an increase in endogenous retrotransposition

As noted above, MOV10 localizes with AGO1 and AGO2 in stress granules and P-bodies [10], sites of translationally-silenced RNPs and mRNA decay, respectively [72]. Likewise, ORF1p is found together with AGO2, and other components of RISC [31], and also closely associates with MOV10 in cytoplasmic granules. We propose that MOV10 is able to recruit L1 RNPs to stress granules (Figure 2), so fating them for silencing and possible degradation by smRNA pathways. Nevertheless, evidence is conflicted for the role of cytoplasmic granules in retrotransposition. Studies to date have dealt with retroviral-like LTR elements and P-bodies only. P-body components are important for retrotransposition of yeast Ty3 retrotransposons [73]. However, P-body disruption increases retrotransposition of mouse IAP elements [74]. Considerably more work is required to elucidate the implications of granule targeting for non-LTR retrotransposition.

Of course, helicases are involved in many other cellular processes, including transcription, pre-mRNA processing, RNA export, translation, RNA storage, RNA decay, and ribosome biogenesis (reviewed in ref. [75]). In turn, retrotransposition is a complex process involving transcription of the full-length L1, RNA transport to the cytoplasm, translation of the bicistronic RNA, formation of an RNP particle followed by its re-import to the nucleus, targeting of the genomic integration site, nicking the DNA bottom strand, priming and reverse transcription, second strand synthesis, and resolution of the integrant. Many mysteries remain concerning this process. At each of these steps helicases could play a role, either promoting or, as we have demonstrated for MOV10, inhibiting retrotransposition. In addition to MOV10, we detected five other RNA helicases associated with the L1 RNP. Compared with MOV10, the effects of their overexpression on cell culture retrotransposition are modest. However, we believe that more detailed investigation of these other helicases, including their knockdown in cells and mouse models, could prove fruitful.

It has been proposed that there is a genetic “arms race” with infecting retroviruses and endogenous retrotransposons, whereby the cell constantly evolves new means to counter infection or transposition. This places selective pressure on the parasitic element, which in turn contrives to evolve counter measures to evade repression [76]. Primate lentiviruses, for example, encode an arsenal of accessory proteins designed to disable host immune factors. These proteins include Virion infectivity factor (Vif), Viral protein X (Vpx), and Viral protein U (Vpu) arrayed, respectively, against cell-encoded APOBEC3G, SAMHD1, and tetherin/BST-2 (summarized in [77], [78]). No viral antagonist of MOV10 has been reported.

One signature of the struggle between host and pathogen is positive selection for alleles that confer fitness benefit. To ascertain if MOV10 shows signs of positive selection, we determined the relative numbers of non-synonymous (dN) and synonymous (dS) nucleotide substitutions per site and dN/dS (ω) ratios over seven primate species using the PAML 4.5 software package [79]. Positive selection would be supported by an excess of non-synonymous amino acid subsitutions (which alter amino acids) relative to synonymous subsitutions, i.e. ω>1. A phylogenic tree for the complete sequences of MOV10 homologs was constructed and ω ratios compared across the primate lineages (Figures S1 and S4). No positive selection was predicted. This is surprising if MOV10 protein is engaged in a coevolutionary arms race with rapidly evolving retroviral proteins. However, strong sequence conservation across primate species is consistent with an essential biochemical role for MOV10. This role might be its function in the RISC complex, although in Drososphila at least, siRNA pathway genes are among the fastest evolving [80]. Lack of positive selection is also expected if a significant function for MOV10 is protection of the genome from LINE1s, the only autonomous active endogenous retroelement. L1s are not rapidly generating new and active variants. Five L1 subfamilies have succeeded each other as a single lineage during the course of hominoid evolution, each replacing the last as the dominant active form [81]. With the exception of a 217-nt long fragment of the coiled-coil domain of ORF1p that shows evidence for positive selection, Boissinot and Furano [82] found strong L1 protein sequence conservation over long periods of time, mediated by clearing of deleterious elements from the genome and purifying selection. This has brought us to the point where, although over half a million defective L1s litter the genome, only about 80 to 100 are considered to be potentially active, most of these members of the youngest L1PA1 subfamily and highly conserved in sequence [83]. If a primary combatant of retrotransposition, the trajectory of primate MOV10 evolution would be expected to fit a “trench warfare” or balancing selection model [84] rather than arms race model, i.e. maintenance of the same beneficial alleles over long periods of time.

Phylogenetic analyses suggest that eukaryote non-LTR retrotransposons predate all LTR retrotransposons, which in turn gave rise to retroviruses through the acquisition of an envelope (env) gene [30], [85]–[87]. With homologs in worms, flies and plants, MOV10 is a member of an ancient subfamily of RNA helicases. Effective against invading retroviruses, MOV10-like proteins may have first evolved to guard the genome against an internal threat.

## Materials and Methods

### Immunoprecipitation and MS sequencing

Cells were lysed in 160 mM NaCl, 50 mM Tris-HCl (pH 7.5), 1 mM EDTA, and 0.25% NP-40, supplemented with protease and phosphatase inhibitor cocktails II and III, vanadyl ribonucleoside complexes, PMSF (Sigma), and RNasin (Promega). Nuclear extracts were prepared with NE-PER Nuclear Protein Extraction Kit (Pierce). RNase inhibitors were omitted from samples treated with 15 µg/ml DNase-free RNase (Roche). FLAG-tagged L1 RNP complexes were immunoprecipitated with anti-FLAG M2 affinity gel (Sigma), and the Johns Hopkins University Mass Spectrometry and Proteomics Facility analyzed immunoprecipitates.

Protein identification of complex samples by LC-MS/MS was performed using an LTQ ion trap MS (Thermo Fisher Scientific) interfaced with a 2D nanoLC system (Eksigent). Peptides were fractionated by reverse-phase HPLC and sequences were identified using Proteome Discoverer and Mascot software (Matrix Science).

IP of V5-tagged proteins from 2102Ep cells (Figure 1G) utilized Protein G Agarose/Salmon Sperm DNA (Upstate).

### Cloning of plasmid constructs

To generate pc-RP-1FH, we introduced by Kunkel mutagenesis [88] tandem FseI-PacI-SgrAI restriction enzyme sites to replace the stop codon of ORF1 L1-RP cloned in the vector pBS KS- (JCC5-RPS). A double FLAG-HA tag was extracted by PCR from the plasmid pOZ-FH-N (gift of Y. Nakatani, Harvard) and cloned between the FseI and SgrAI sites. The tagged L1 was then inserted between NotI/blunted SacII sites of pcDNA6 myc/his B (Invitrogen).

ORF1 TAP-tagged L1 and ORF1-GFP-L1-RP constructs have been described [31], [39]. FLAG-MOV10L was a gift from R. Frost and E. Olsen (University of Texas Southwestern Medical Center), mRFP-Mov10 was from R. Burdick and V. Pathak (NCI, NIH), pcDNA3.1-V5-His-MOV10 wild-type and helicase motif mutants Mut-I-VI were from Y.-H. Zheng (Michigan State University, East Lansing), and 6myc-p72 (full-length DDX17 isoform 1) was a gift from R. Janknecht (University of Oklahoma; [89]). Ultimate pENTR ORF clones for MOV10 (Cat. no. IOH4005), CBX7 (IOH54861), CBX8 (IOH27823), DDX17, isoform 2 (IOH1750), DDX21 (IOH46173), DDX39A (IOH3477), fibrillarin (IOH14368), and YBX1 (IOH3666), were V5-tagged on their N-termini by shuttling them into pcDNA3.1/nV5-DEST using Gateway technology (Invitrogen). DDX5 (NM_004396) and DHX9 (NM_001357) were amplified by PCR from the CytoTrap XR human testes cDNA library (Stratagene), cloned in the Gateway pDONR vector, and shuttled into pcDNA3.1/nV5-DEST. For its use in *neoI* retrotransposition assays, MOV10 cDNA was recloned in a modified Gateway system vector lacking the *neo* gene (a gift from H. Zhu, Johns Hopkins).

RNAi Consortium shRNA library (Open Biosystems) clones were shGAPDH (TRCN0000221342), shE1 (TRCN0000049978), shE2 (TRCN0000049981), and shE3 (TRCN0000049980). These were packaged in lentiviral particles in 293T cells by standard methods.

99 PUR RPS EGFP, 99 PUR JM111 EGFP, Alu neo^Tet^ and SVA.2 retrotransposition reporter constructs have been described [28], [34], [49]. ORF1-GFP L1-RP was reported [39], and ORF1 GFP RRM L1-RP introduced the following ORF1 mutation into ORF1p-GFP L1-RP: 155-RPNLRLIGVPE-165> AAAAAAAGVAA.

### Cell culture and retrotransposition assay

Human embryonic kidney HEK293T cells, HeLa-HA cells, and 2102Ep cells were grown in DMEM medium with 10% FBS (Hyclone), GlutaMax, and Pen-strep (Invitrogen). All transfections used FuGENE HD (Promega) reagent. 2102Ep and HeLa-HA cells were gifts from P. K. Andrews (U. of Sheffield, United Kingdom) and J.V. Moran (U. of Michigan), respectively.

The EGFP L1 cell culture retrotransposition assay was conducted as previously described [34]. 2.5×10^5^ HeLa or 293T cells/well were seeded in 6-well dishes. The following day, 1.0 µg of 99 PUR RPS EGFP, a plasmid containing L1-RP and EGFP retrotransposition reporter cassette, was cotransfected together with 0.5 µg empty vector (pcDNA3, Invitrogen) or test plasmid. All transfections were in quadruplicate. Five days post-transfection, cells having a retrotransposition event and hence expressing EGFP were assayed by flow cytometry. Gating exclusions were based on background fluorescence of plasmid 99 PUR JM111 EGFP, an L1 construct containing two point mutations in ORF1 that completely abolish retrotransposition [47]. Within each experiment, results were normalized to fluorescence of 99 PUR RPS EGFP cotransfected with pcDNA3. Transfection efficiency and toxicity was monitored by cotransfection of CEP-EGFP, a plasmid that constitutively expresses green fluorescent protein, and empty vector or helicase constructs, followed by FACs analysis after four days.

Alu retrotransposition assays were carried out essentially as described in Dewannieux et al. [49]. Retrotransposition construct Alu-*neo^Tet^* was cotransfected with pcDNA3 or retrotransposition driver plasmids pCEP 5′UTR ORF2 No Neo [50] (a gift from J.L. García-Pérez, Pfizer-University of Granada), or pcDNA L1-RP [28]. Eighteen hours post-transfection, HeLa-HA cells were expanded from six-well plates to T_75_ flasks, and four days later selection for retrotransposition events with 600 µg/ml of G418 was begun. After 15 days of selection, cells were fixed, stained with Giemsa, and colonies were counted.

SVA retrotransposition assays were conducted as previously described. SVA.2 *mneoI* was cotransfected with both pCEP.ORF2 and pcDNA.ORF1-RP in HeLa-HA cells, expanded to T_75_ flasks, and selected with G418 as described above for the Alu assay. SVA.2 EGFP was cotransfected in 293T cells together with pCEP.ORF2 or pcDNA.ORF1-RP, and retrotransposition was examined by FACs after 5 days [28].

### Immunofluorescence and Western blotting

Immunofluorescence techniques have been described [31]. Purified AH40.1 polyclonal antibody against ORF1 protein was a gift from T. Fanning (Armed Forces Institute of Pathology, MD) and M. Singer (Carnegie Institution of Washington). Polyclonal affinity-purified antibodies α-ORF1 (318–338) and α-ORF2-N (154–167) were described in Goodier et al. [38]. α-β-tubulin (E7) was from the Developmental Studies Hybridoma Bank (U. of Iowa). Commercial antibodies included ms α-V5-tag (Invitrogen), rb α-Myc-tag 71D10 (Cell Signaling), rb α-MOV10 (Proteintech), and rb α-MOV10(111–125) (Sigma). Donkey Cy3-, Cy5-, DyLight 488-, or DyLight 549-conjugated, and HRP-conjugated secondary antibodies were from Jackson ImmunoResearch Laboratories.

Western blot antibody incubation was in blocking solution (PBS/0.05% Tween 20/5% dry milk) overnight at 4°C. Membranes were washed 3× for 15 min with PBS/0.05% Tween 20, incubated with secondary antibody at room temperature in blocking solution for 2 hr and washed again. Detection used SuperSignal West Pico Chemiluminescent Substrate (Pierce).

### Reverse transcriptase assay and RT–PCR

Reverse transcriptase analysis followed the LEAP protocol of Kulpa and Moran [35]. Primers used were:

3′RACE adapter NV: GCGAGCACAGAATTAATACGACTCACTATAGGTTTTTTTTTTTTVN

3′RACE outer: GCGAGCACAGAATTAATACGACT

aORF2-end1: CACCGCATATTCTCACTCATAGG

Two µl of IP sample were added to each cDNA extension reaction. PCR amplification of cDNA utilized Expand Long Template PCR System (Roche). Products were separated on 2% agarose gels.

For RT–PCR analyses, immunoprecipitates were directly treated with TURBO DNase (Invitrogen), and cDNA generated using iScript cDNA Synthesis Kit (Bio-Rad). Standard PCR reactions used the Expand Long Template PCR System (Roche) and the following primers: bORF2-end2, GATGAGTTCATATCCTTTGTAGGG [35]; and BGHPOLYAREV, GGGAGTGGCACCTTCCAGGGTC.

### L1 promoter methylation analysis

Bisulfite analysis was performed exactly as described [90], [91] (Figure S3). The region amplified for analysis spanned 363-bp of the L1 5′UTR and contained 20 CpG dinucleotides. PCR products were subcloned (TOPO TA Cloning Kit, Invitrogen) and analyzed with online software (QUantification tool for Methylation Analysis, QUMA; quma.cdb.riken.jp [92]). A few amplified sequences were not members of the young L1Hs/L1PA1 family and were excluded from the analysis. Significance or methylation differences were examined with Fisher's Exact Test.

### Sequence analysis

Protein sequences of Figure S1 were aligned with WebPRANK (http://www.ebi.ac.uk/goldman-srv/webPRANK/; [93]) and hand-aligned with Jalview (http://www.jalview.org; [94]) (Figures S1 and S4). The evolutionary tree was calculated using the Maximum Likelihood method from CODEML of the PAML 4.5 software package [79]. The global synonymous changes per site (dS)/nonsynonymous changes per site (dN) ratios for the tree were calculated by the free-ratio model from CODEML (F61 model of codon frequencies). The free-ratio model allows the dN/dS ratio to be independent on all lineages. The tree with the highest log likelihood is shown Figure S4B. The initial tree for the PAML was obtained through evolutionary analyses in MEGA5 [95]. Analysis for positive selection in the MOV10 gene used PAML 4.5 [79] (Figure S4).

## Supporting Information

Figure S1 Alignment of primate MOV10 protein sequences. Dot indicates identity to the human sequence, and dash indicates a gap. Sequences were obtained from either Genbank or ENSEMBL databases with the following accession numbers: Homo sapiens (NM_001130079.1), Chimp (Pan troglodytes, XM_513630.3), Gorilla (Gorilla gorilla; ENSGGOT00000002753), Orangutan (Pongo abelii; ENSPPYT00000001228), Gibbon (Nomascus leucogenys; ENSNLET00000005378), Rhesus macaque (Macaca mulatta; ENSMMUT00000021988); Marmoset (Callithrix jacchus; XP_002751280.1), and Bushbaby (Otolemur garnettii; ENSOGAT00000014085). Unaligned insertions and deletions in the gibbon sequence are likely assembly errors. Conserved helicase motifs are blocked in pink, and residues altered in MOV10-V5-His_6_ mutant constructs (Figure 3H) are underlined in red [16].
(TIF)

Figure S2 Construct pc-L1-1FH-EGFP is retrotransposition-competent, but at a reduced level compared with a similar construct lacking the FLAG-HA tag on ORF1 (pc-L1-RP-EGFP). The EGFP reporter cassette [34] was introduced into an AleI restriction enzyme site in the 3′ UTR of tagged and untagged L1 constructs, and assayed for retrotransposition in 293T cells [34].
(TIF)

Figure S3 Methylation analyses of the 5′ UTR promoter of endogenous L1 elements show no effect of MOV10. (A) The individual methylation status of 36 L1 sequences in the presence (right) or absence (left) of V5-MOV10 protein. Open circles, closed circles and lines represent unmethylated, methylated, and mutated CpG positions, respectively. (B) The percentage of methylation of the 20 CpG residues in the absence (white) or presence (black) of V5-MOV10. Applying Fisher's Exact Test, no significant effect of ectopic MOV10 expression on L1 5′ UTR methylation status was found. CpG residues are numbered according to the retrotransposition-competent element L1.3 (accession number L19088.1).
(TIF)

Figure S4 Analysis of primate MOV10 genes for positive selection using maximum likelihood estimation and PAML4 software. (A) Pair-wise comparisons of dN and dS among the eight primate MOV10 sequences shown in Figure S1. The values for dN and dS were calculated by yn00 from the PAML 4.5 software package [79]. The diagonal line indicates ω (dN/dS) = 1. (B) Highest log likelihood phylogenetic tree of the primate MOV10 sequences. ω values (dN/dS) are shown above each branch. No branches were determined to be under positive selection. (C) Random-sites models, log-likelihood values (lnL), and positively selected sites (PSS). Results for both F61 and F3X4 codon substitution models are shown. Both models gave similar results. np, number of parameters in the distribution. NEB, Naive empirical Bayes. (D) Model comparisons testing for departures from neutrality and positive selection. None were significant at p<0.01.
(TIF)

Table S1 RNA helicase proteins identified after LC-MS/MS and Mascot protein/peptide analyses of immunoprecipitated L1 RNPs.
(TIF)
