# Supplementary figures and images for: MOV10 RNA Helicase Is a Potent Inhibitor of Retrotransposition in Cells
Source: PLoS Genet. 2012 Oct 18;8(10):e1002941. doi: 10.1371/journal.pgen.1002941 (PMC3475670; doi:10.1371/journal.pgen.1002941)

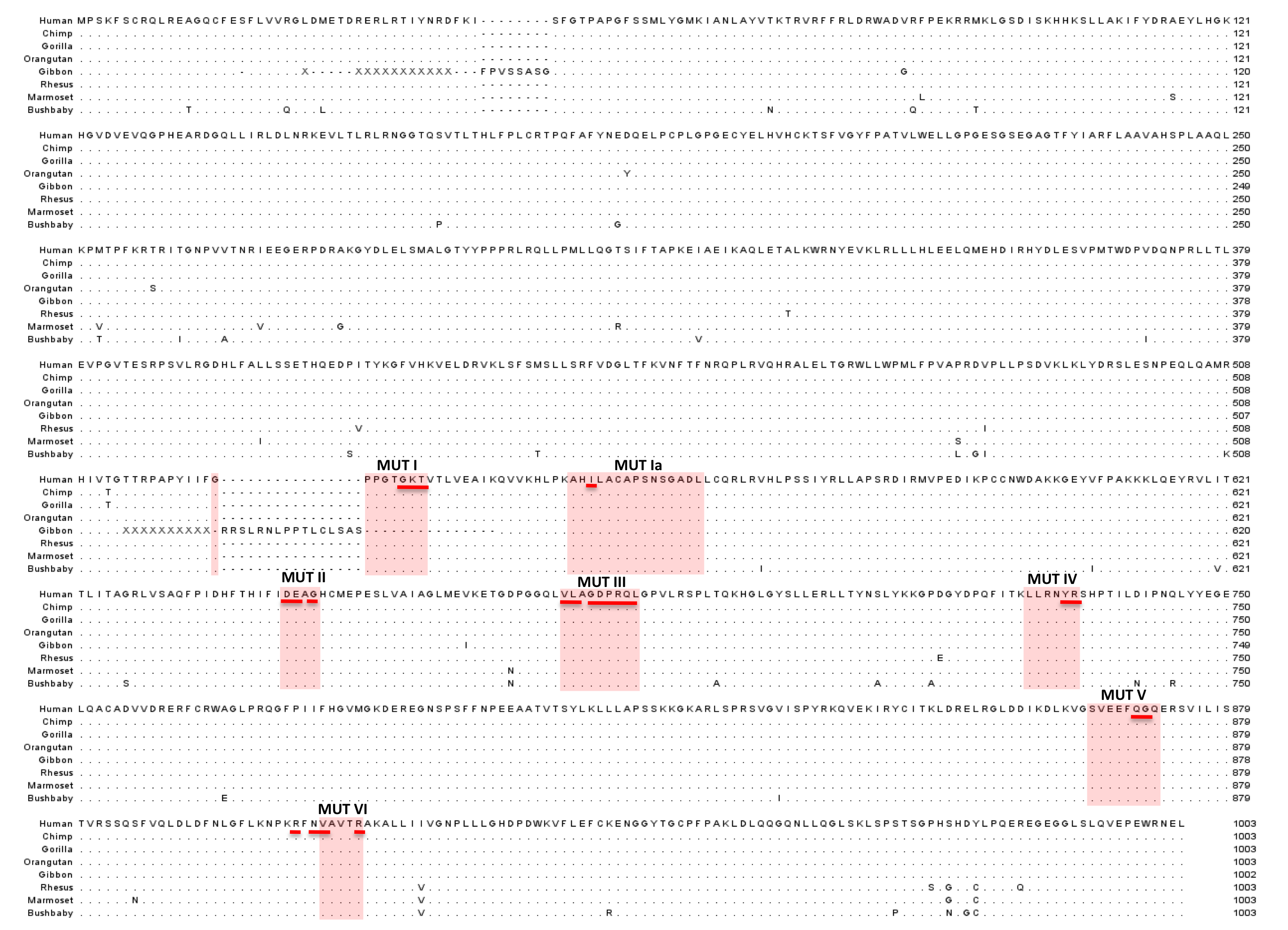

Supplement: Figure S1 — Alignment of primate MOV10 protein sequences. Dot indicates identity to the human sequence, and dash indicates a gap. Sequences were obtained from either Genbank or ENSEMBL databases with the following accession numbers: Homo sapiens (NM_001130079.1), Chimp (Pan troglodytes, XM_513630.3), Gorilla (Gorilla gorilla; ENSGGOT00000002753), Orangutan (Pongo abelii; ENSPPYT00000001228), Gibbon (Nomascus leucogenys; ENSNLET00000005378), Rhesus macaque (Macaca mulatta; ENSMMUT00000021988); Marmoset (Callithrix jacchus; XP_002751280.1), and Bushbaby (Otolemur garnettii; ENSOGAT00000014085). Unaligned insertions and deletions in the gibbon sequence are likely assembly errors. Conserved helicase motifs are blocked in pink, and residues altered in MOV10-V5-His6 mutant constructs (Figure 3H) are underlined in red [16]. (TIF) [file pgen.1002941.s001.tif]

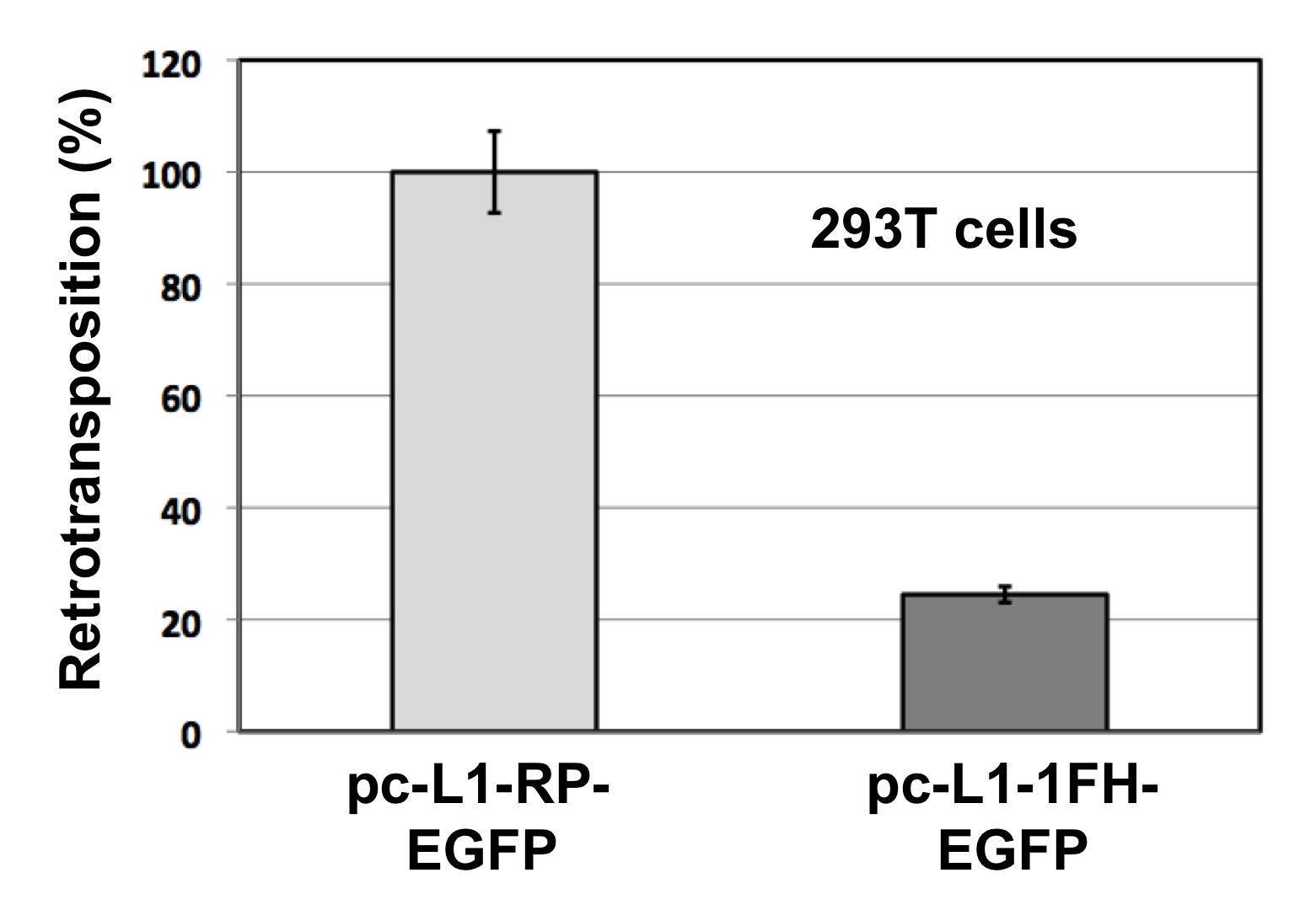

Supplement: Figure S2 — Construct pc-L1-1FH-EGFP is retrotransposition-competent, but at a reduced level compared with a similar construct lacking the FLAG-HA tag on ORF1 (pc-L1-RP-EGFP). The EGFP reporter cassette [34] was introduced into an AleI restriction enzyme site in the 3′ UTR of tagged and untagged L1 constructs, and assayed for retrotransposition in 293T cells [34]. (TIF) [file pgen.1002941.s002.tif]

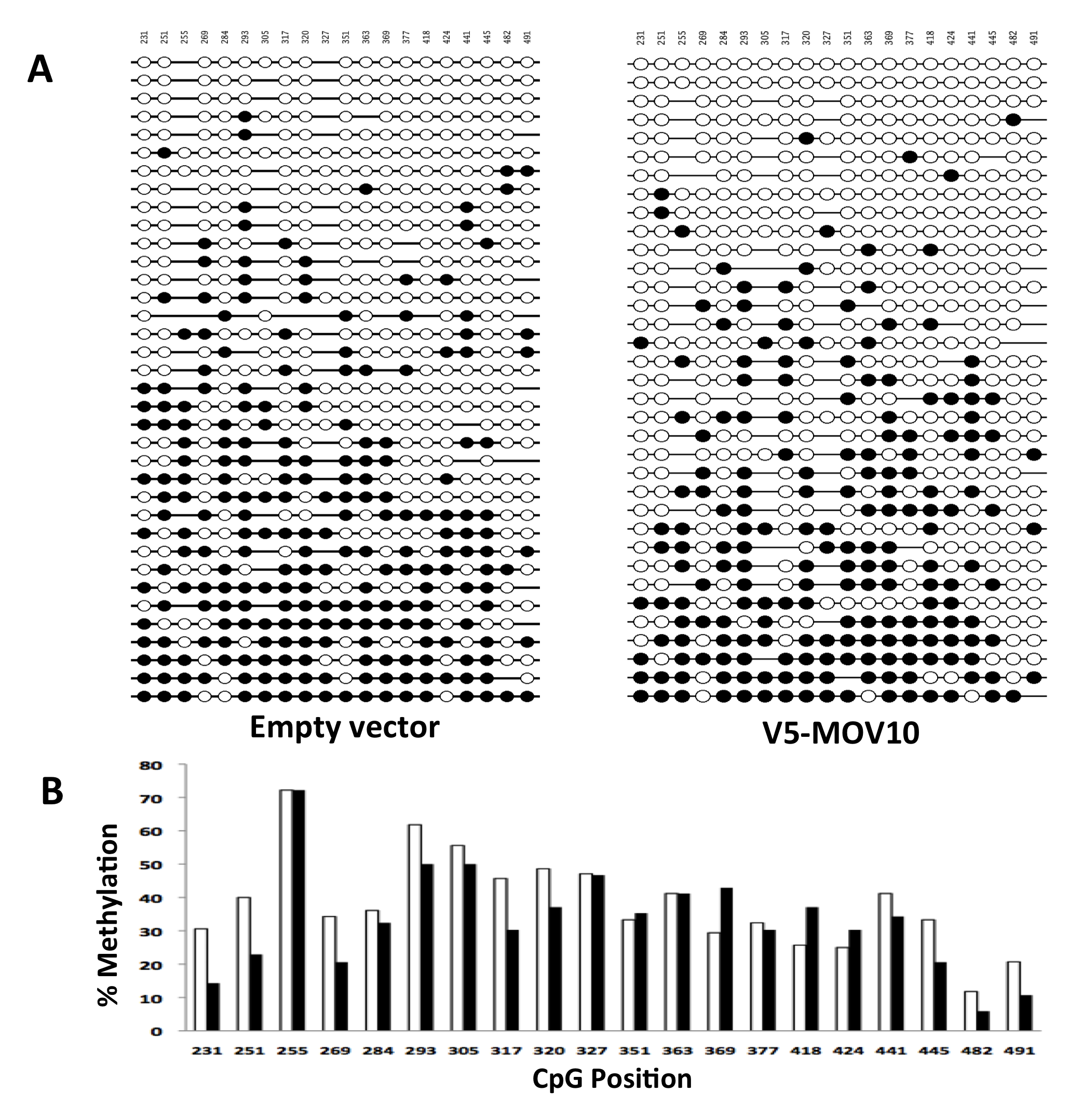

Supplement: Figure S3 — Methylation analyses of the 5′ UTR promoter of endogenous L1 elements show no effect of MOV10. (A) The individual methylation status of 36 L1 sequences in the presence (right) or absence (left) of V5-MOV10 protein. Open circles, closed circles and lines represent unmethylated, methylated, and mutated CpG positions, respectively. (B) The percentage of methylation of the 20 CpG residues in the absence (white) or presence (black) of V5-MOV10. Applying Fisher's Exact Test, no significant effect of ectopic MOV10 expression on L1 5′ UTR methylation status was found. CpG residues are numbered according to the retrotransposition-competent element L1.3 (accession number L19088.1). (TIF) [file pgen.1002941.s003.tif]

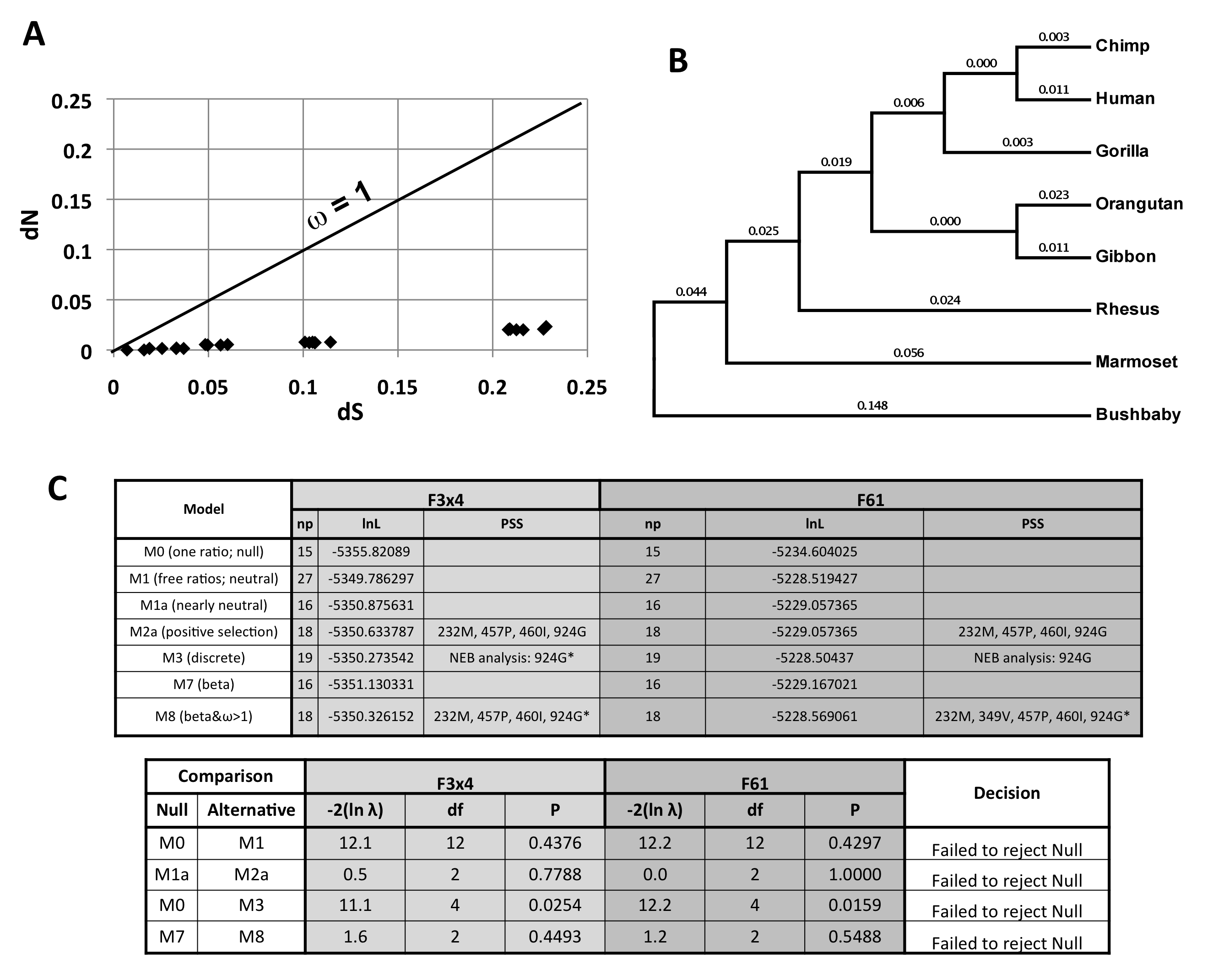

Supplement: Figure S4 — Analysis of primate MOV10 genes for positive selection using maximum likelihood estimation and PAML4 software. (A) Pair-wise comparisons of dN and dS among the eight primate MOV10 sequences shown in Figure S1. The values for dN and dS were calculated by yn00 from the PAML 4.5 software package [79]. The diagonal line indicates ω (dN/dS) = 1. (B) Highest log likelihood phylogenetic tree of the primate MOV10 sequences. ω values (dN/dS) are shown above each branch. No branches were determined to be under positive selection. (C) Random-sites models, log-likelihood values (lnL), and positively selected sites (PSS). Results for both F61 and F3X4 codon substitution models are shown. Both models gave similar results. np, number of parameters in the distribution. NEB, Naive empirical Bayes. (D) Model comparisons testing for departures from neutrality and positive selection. None were significant at p<0.01. (TIF) [file pgen.1002941.s004.tif]

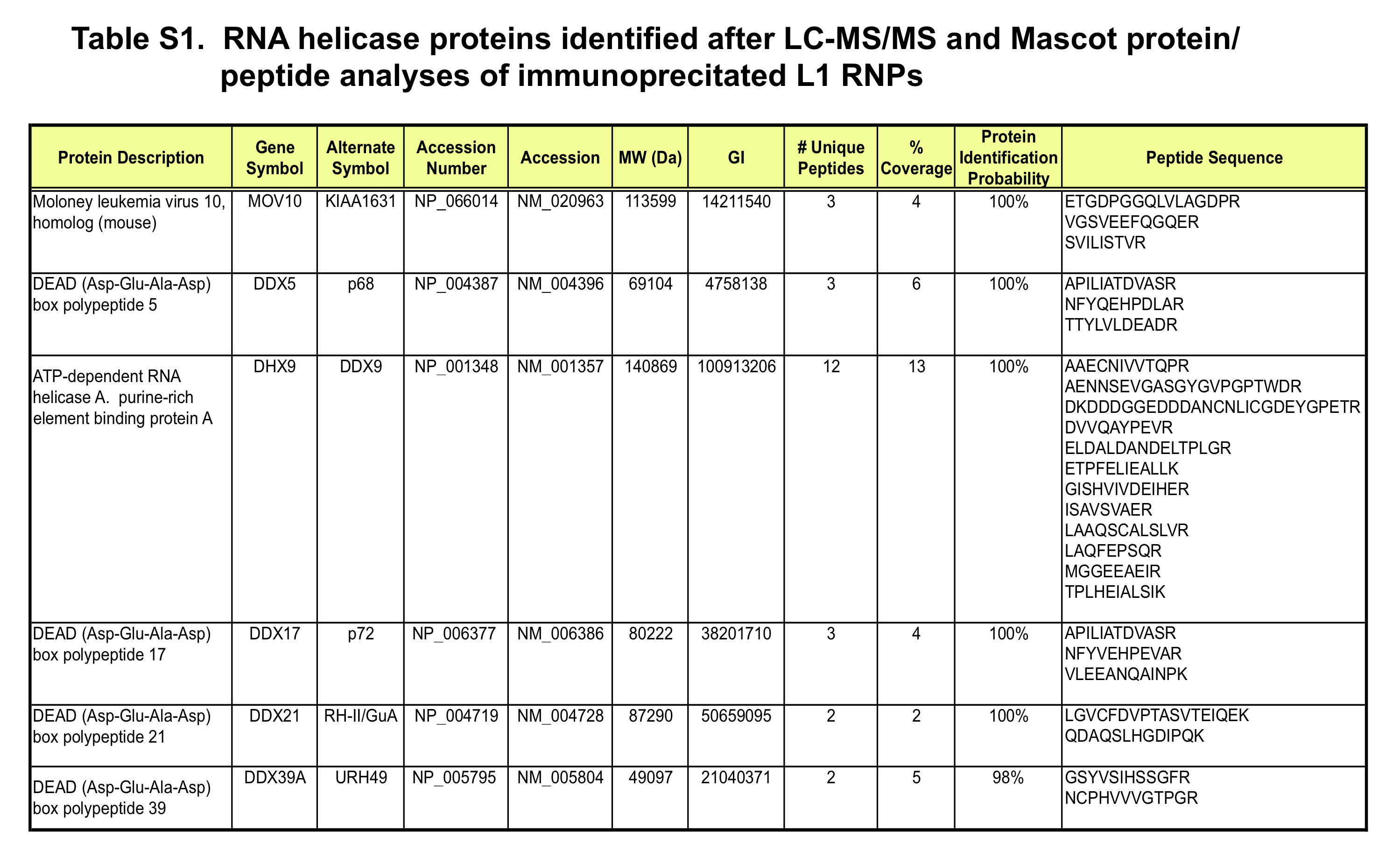

Supplement: Table S1 — RNA helicase proteins identified after LC-MS/MS and Mascot protein/peptide analyses of immunoprecipitated L1 RNPs. (TIF) [file pgen.1002941.s005.tif]
